# Analysis of the Machine-Specific Behavior of Injection Molding Machines

**DOI:** 10.3390/polym16010054

**Published:** 2023-12-22

**Authors:** Julia Knoll, Hans-Peter Heim

**Affiliations:** Institute of Material Engineering, Polymer Engineering, University of Kassel, 34125 Kassel, Germany; heim@uni-kassel.de

**Keywords:** injection molding, machine-specific behavior, process analysis, machine fingerprint

## Abstract

The performance of an injection molding machine (IMM) influences the process and the quality of the parts manufactured. Despite increasing data collection capabilities, their machine-specific behavior has not been extensively studied. To close corresponding research gaps, the machine-specific behavior of two hydraulic IMMs of different sizes and one electric IMM were compared with each other as part of the investigations. Both the start-up behavior from the cold state and the behavior of the machine at different operating points were considered. To complement this, the influence of various material properties on the machine-specific behavior was investigated by processing an unreinforced and glass-fiber-reinforced polyamide. The results obtained provide crucial insights into machine-specific behavior, which may, for instance, account for disparities between computer fluid dynamic (CFD) simulations and experimental results. Furthermore, it is expected that the description of the machine-specific behavior can contribute to transfer knowledge when applying transfer learning algorithms. Looking ahead to future research, it is advised to create what is referred to as a “machine fingerprint”, and this proposal is accompanied by some preliminary recommendations for its development.

## 1. Introduction

**Figure 1:**
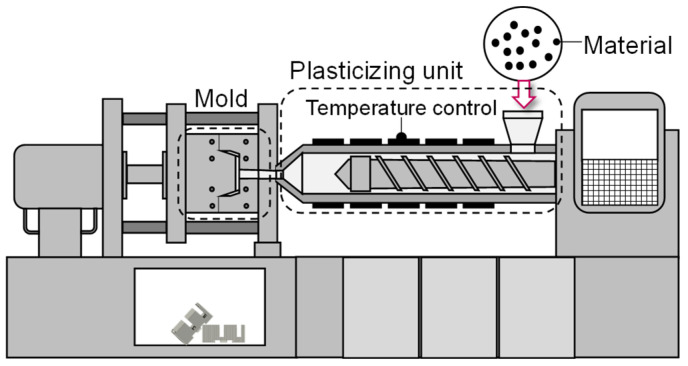
Injection molding machine with exchangeable mold.

The typical setup of an IMM is presented in Figure 1. During the discontinuous process, the material is fed to the plasticizing unit, in which the granules are plasticized. The melting is achieved by the temperature control of the attached heating bands as well as the rotational movement of the screw. To mold the viscous melt, the machine performs a feed movement of the screw to inject the plastic into the cavity of the mold. Thereby, the feed rate as well as the amount of plastic injected are determined by the flow rate and the start and end position of the screw during injection, which are specified by the machine operator.

**Figure 2:**
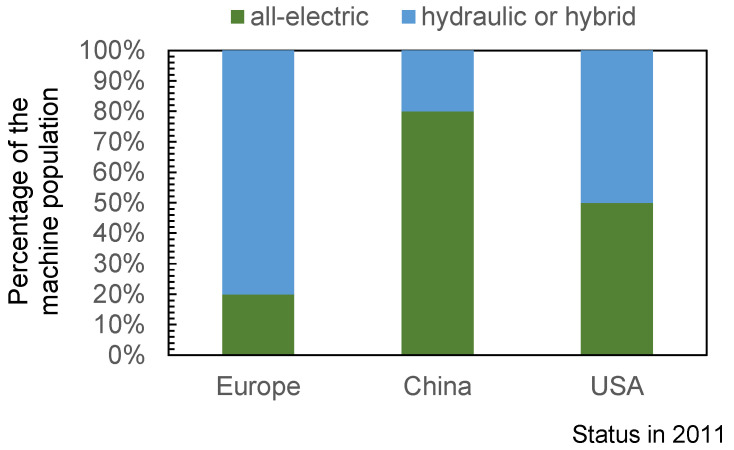
Percentage of all-electric and hydraulic or hybrid injection molding machines on the European, Chinese, and American markets; data cited in [1].

The advantage of injection molding is that the injection mold is exchangeable, so that different parts can be produced on one machine, or in other words, one mold can be operated on different machines. This offers plastics processing companies a certain flexibility in their choice of the material–mold–machine combination (MMMC) if compatibility is given. However, every MMMC results in a different production scenario which means that the processes have to be set up anew. This is due to the fact that the machine influences the process as well as the process result by its specific behavior. On the market, there are various types of injection molding machines available, including those equipped with electric variable displacement pumps, hydraulic machines with variable displacement pumps, hydraulic machines with variable motors, and all-electric machines. A study for Euromap (www.euromap.org, accessed on 15 October 2023) in 2011 has shown that the percentage of machine population is region-specific, as can be seen in Figure 2 [1]. The study showed an increase in electric machines in Europe, so it can be assumed that the current market population of electric machines is higher.

Due to the widespread use of electric as well as hydraulic machines, both types of drive were considered within the scope of these investigations. The general influences of the injection molding machine on the process are described below.

### Influence of the Machine during the Injection Molding Process

The control of the injection molding machine influences the accuracy of the screw position, which in turn affects dosing, injection, and holding pressure. If too little melt is injected, short shots, sink marks, and warpage can occur [2]. Conversely, if the switchover is too late, the cavity can be overloaded, and over-injection and extreme cavity pressures may occur [2,3]. To optimize the process control, the concept of a cross-phase control strategy was presented [4]. By using a model-based predictive controller, no switchover is required due to a smooth control of the cavity pressure. For this approach, an optimum cavity pressure trajectory must be defined for each production scenario. Consequently, such control strategies require adaption to the specific machine behavior.

Likewise, the temperature control of the machine has an influence on the process and the resulting part quality: The material temperature is caused, on the one hand, by the shearing of the material due to the rotational movement of the screw and, on the other hand, by the temperature control of the barrel heating bands as well as the nozzle [5,6]. The temperature control is regulated using PID controllers to ensure that the temperature remains as constant as possible from cycle to cycle. Depending on the controller, the temperature dynamics of the machines can differ. In their study, Yao et al. [7] discuss the effects of temperature fluctuations, which can result in inhomogeneous melt temperatures. Specifically, significant temperature fluctuations and the overshooting of the control have a noticeable impact on the melt’s flow characteristics and, consequently, part quality. On one hand, there is the potential for polymer degradation when processing thermally sensitive materials, and on the other hand, when temperatures are too low, flow resistance can increase. In both scenarios, temperature fluctuations negatively affect the quality of the parts.

Besides the control, the age of the machine has a significant influence [8]. Depending on the machine’s period of use, signs of wear and tear may occur. The wear of the non-return valve has an influence on the ejection behavior [9,10,11] and the process stability [12,13]. A comparison of different machine types in [8] has shown that servo-electric machines can have a more precise control of the screw position, but the reproducibility of the part quality was worse [8]. An analysis of the machine behavior based on process data and an investigation of the material influence with identical machine configurations were not part of the investigations in [8] and still need to be carried out.

In addition to the machine-dependent influencing factors, the material itself has an impact. The flow behavior of the melt influences the back pressure during injection, which in turn affects the position control of the screw [3]. Thus, different material types or additives such as fibers can influence machine behavior, whereby even small variations between material batches of nominally identical materials can cause significant variations [11,14].

In addition to the influences described, several disturbance variables make the control of production difficult [15,16]. To ensure the production of good parts during the continuous operation of the machine, a variety of supervised machine learning methods can be used to model part characteristics as a function of machine setting parameters. Neural networks, fuzzy logic, and support vector machines have shown good results in modeling individual part characteristics as a function of the operating point of the IMM [17,18,19,20,21]. Process parameter curves, such as injection pressure, can be applied to explain process variations, i.e., in part weight [22]. In addition to offline models, online models have been developed that enable the prediction of part quality during ongoing processes [23,24,25]. This included the creation of a digital twin, which can be used to recommend a new operating point in order to achieve the specified quality [24,25]. Nevertheless, the validity of such models is always limited to the MMMC which was used for data acquisition. Using transfer learning approaches, machine learning models such as neural networks can be pre-trained with data from one or more MMMCs and applied and re-trained on to another process (which is “unknown” to the model) [26]. Such models offer the possibility of transferring already trained process correlations to another MMMC. However, the accuracy of such models depends on the similarity of the processes with respect to the correlations represented in the data. The similarity as well as the process correlations are influenced by the machine-specific behavior. To characterize this machine-specific behavior, a so-called machine fingerprint could be derived, which describes, for example, the typical characteristics of the control behavior and the properties of the injection molding machine that influence the process sequence and the process result.

Another way of modeling relationships between the operating point and the part characteristics can be done via CFD simulation. Software packages such as Moldflow^®^ (Autodesk Moldflow Insight Version 2021.2) and Moldex 3D^®^ (Moldex 3D 2022 R2) model the process from the moment the melt enters the mold. To reduce the simulation effort for modeling the process, transfer learning algorithms can be used. They are able to model the process by using simulation data describing the process of one or more MMMCs and transferring the knowledge to another MMMC while fewer simulation data for the new MMMC are needed [27]. In order to be able to apply appropriate approaches in practice, it is assumed that the simulation and the experiment correspond. But the machine-specific behavior of the screw during the metering and ejection of the melt is not included in the simulation computations so far. Accordingly, studies [28] have shown, that the accuracy of the simulation and the real process depends on both the machine and the setting parameters. To what extent the machine influences the discrepancy between experiment and simulation (which neglects the machine behavior) is unknown so far. And also in [27] it is recommended that “the combination of process, material and part influences on the injection molding process when modelling the processes needs to be the near objective as this could lead to the development of a machine-specific modelling approach for machine parameter optimization”. A description of the machine-specific behavior is still pending.

Summing up, there are two ways of process modeling, machine learning and CFD. In both cases, the machine-specific behavior is relevant for the accuracy of the models, when comparing the calculated and experimental data. Until now, there are no methods that include the machine behavior in modeling, e.g., by defining a machine fingerprint. Especially in the context of industry 4.0 and digitalization, machine-specific behavior is of interest. To examine the reasons for the deviation between simulation and experiment in more detail and to provide the basis for generating the machine fingerprint, the behavior of three different injection molding machines was compared with each other as part of these investigations. Therefore, the following research questions were focused on:

- How does the machine-specific behavior of the machine influence the process when it is started from a cold state?
- How much do the processes differ when using different machines? What influence do the operating points have in each case?
- Do changes in the material characteristics affect the machine-specific behavior?

In the course of these investigations, partially filled parts were produced in order to visualize the precision of the machines when controlling the switchover point. To evaluate the injection phase, the ejected polymer mass was used. In addition, the time series of the process parameters flow rate, screw volume, and injection pressure were studied for a detailed analysis of the influence of the machine and its control on the process sequence.

To evaluate the influence of the machines in dependence on the material and mold, three different series of experiments were carried out (see Table 1). The respective machines, materials, molds, and experimental designs are described in the following section.

During Part A of these investigations, the start-up behavior of three different injection molding machines was analyzed, with comparable conditions being created for all production scenarios. To investigate the machine-specific behavior at different operating points, an experimental design was carried out by using three different injection molding machines during Part B of these studies. During the experiments, all other possible influencing variables were eliminated or kept constant (closed mold, same material with the same preconditioning). To further investigate the influence of the material and the back pressure on the precision of the machine and the ejected polymer mass, different materials with closed and opened molds were used during the third part of the experiments. In this way, the influence of the material viscosity and the back pressure was to be qualitatively evaluated.

In addition, as a forward-looking aspect for future research, the findings were used to evaluate which variables should be taken into account for the development of a machine fingerprint.

## 2. Materials and Methods

Two hydromechanical (HM) IMMs with the type “Arburg 320 C Golden Edition” (A) and “Arburg Allrounder 470 S” (B) and one electromechanical (EM) IMM, “Arburg Allrounder 520 E” (C), of the company Arburg GmbH + Co KG (Loßburg, Germany) were used for the investigations. Further information about the machines can be found in Table 2.

The machine-specific behavior was investigated for a constant operating point during start-up (see Table 3) and also for various setting parameters. Thus, the influence of the machine’s setting parameters on the dynamic behavior of the machine and the ejected polymer mass was studied. To study the behavior at different operating points, the injection flow rate (Q_inj_), the dosing (V_d_), and switchover volume (V_s_) as well as the temperature profile of the cylinder heating zones (represented by the max. cylinder temperature T_c_) were varied according to Table 4 (Part B) and Table 5 (Part C). In supplement to the max. cylinder temperature T_c_, Table A1 lists the setting values for the individual cylinder heating zones when using machines A, B, and C. When the nozzle temperature T_c_ was increased to 260 °C or 270 °C, the values listed in Table A1 were increased for each temperature zone by 10 °C or 20 °C, respectively. Despite the different number of cylinder heating zones, the aim was to achieve the most similar temperature profiles possible along the cylinder. During Parts B and C, the machine settings were adjusted via the machine’s control panel. To ensure the reproducibility of the process, six test specimens were manufactured for each parameter configuration. After adjustments of T_c_, approximately 30 test specimens for each parameter configuration were manufactured before producing the test specimens. This was done to establish thermal stability and ensure stable processing conditions. For the experiments during Parts A and B, a mold for producing tensile test specimens according to DIN EN ISO 527-2 [29] type 1A was utilized. For the experiments during Part C, a mold for producing test specimens in accordance with the standard UL94 with a length of 160 mm, a width of 20 mm, and a thickness of 4 mm was used. To investigate the influence of the machine control during injection, the dosing and switchover volume was set so that partially filled parts were produced. 

When selecting the experimental design for Part B and Part C, simple factorial test plans with two factor levels were chosen. Prior to the experiments, sampling was carried out and the limits of the experimental design factors were determined. The machine’s capabilities were taken into account when selecting the limits of the flow rate and the volumetric filling of the parts when selecting the dosing and switchover volumes. The factor levels of the maximum cylinder temperature were selected identically for both Part B and Part C, as this was derived from the specifications provided by the material data sheets. A central point (No. 5 in Table 4) was also added to the experimental design in Part B, which corresponds to the machine setting in Part A. The dosing and switchover volumes were considered as one factor, as both parameters were always varied by the same amount to achieve the same volumetric filling. Due to the different mold geometries, different settings of the dosing and switchover volumes were chosen for the experimental designs in Parts A and B versus Part C. 

The remaining parameters, which were not varied during the experiments, are shown in Table 6.

A non-reinforced polyamide 6 (PA) and a reinforced polyamide 6 with a glass fiber content of 30% (PAGF30) were used for the experimental work, which were provided by the company BASF (Ludwigshafen, Germany). The main properties describing the materials are shown in Table 7.

Prior to the injection molding process, the materials were dried using an air dryer (TORO-systems TR-Dry-Jet EASY 15, provided by “GfK Gesellschaft für angewandte Kunststofftechnik”, Igensdorf, Germany) until a moisture of approx. 0.15% was achieved.

Continuous data recording throughout the entire experiment was implemented by using the OPC UA interfaces of the machines to obtain time series data of the injection pressure, flow rate, cavity pressure, and screw volume. For the weighing of the manufactured specimens, the scale Sartorius Practum 224-1S of the company Sartorius AG (Göttingen, Germany) with a resolution of 0.1 mg was used.

## 3. Results and Discussion

### 3.1. Part A: Evaluation of the Start-Up Behavior Based on the Ejected Polymer Masses

**Figure 3:**
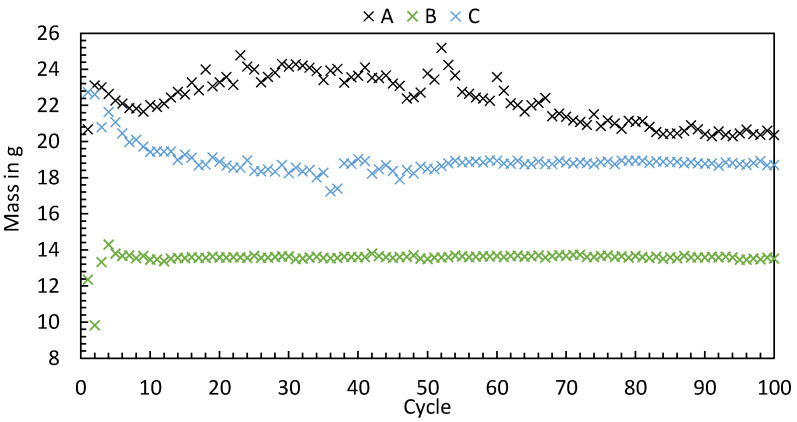
Weight of the polymer masses ejected by three different injection molding machines with identical process conditions.

To investigate the start-up behavior, every machine was parameterized according to Table 4, and the ejected polymer masses of the first 100 cycles after switching on the machine were evaluated. The measured values, visualized in Figure 3, show that the machines perform differently during start-up. The ejected polymer masses differ significantly from one another. Machine A exhibited the longest transient phase, which can be seen from the fact that the weight decreased at the beginning, subsequently increased, and then decreased again until equilibrium was reached after approximately 85 cycles. Machine B, in contrast, exhibits equilibrium of the ejected polymer mass much earlier. In the first cycles, an increase and decrease in polymer mass can be seen, with equilibrium having already been reached after 7–8 cycles. Machine C, the all-electric machine, showed a decrease in ejected polymer mass at the beginning, with equilibrium only being reached from cycle 55. 

The results show that the start-up behavior of the machines differs. The reasons for the differences in start-up behavior cannot be deduced from the data recorded. But neglecting this behavior can lead to erroneous assumptions when conducting experiments or modeling the process. Figure 3 shows that the machine-specific start-up behavior influences the mass of the parts manufactured depending on the number of cycles. Depending on the part respectively to the cycle number under consideration for process evaluation, the process may still be in a transient state. This can lead to a misinterpretation of the correlation between the process setting and the part characteristics. A data-driven evaluation of the start-up behavior based on the high-resolution time series of the process parameters, as already presented in [30], offers the possibility to provide the machine operator the information about the point at which a reproducible process is present without measuring each individual part.

### 3.2. Part B: Analysis of the Machine-Specific Behavior of Three Different IMMs at Varying Operating Points

**Figure 4:**
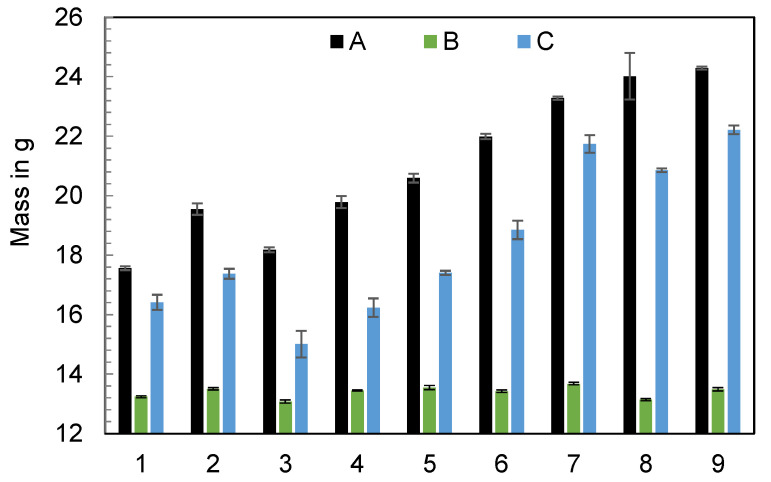
Ejected polymer mass using the three injection molding machines A, B, and C, by varying the machine’s setting parameters (n = 6).

In addition to the start-up behavior, the behavior of the machine in a steady state is of interest. For this purpose, the influence of the machine type on the polymer mass depending on the machine settings was studied. Therefore, the experimental design from Table 4 was used to set different operating points of the machine. Immediately after each cycle, the mass of the ejected polymer was measured, which is presented in Figure 4. 

**Figure 5:**
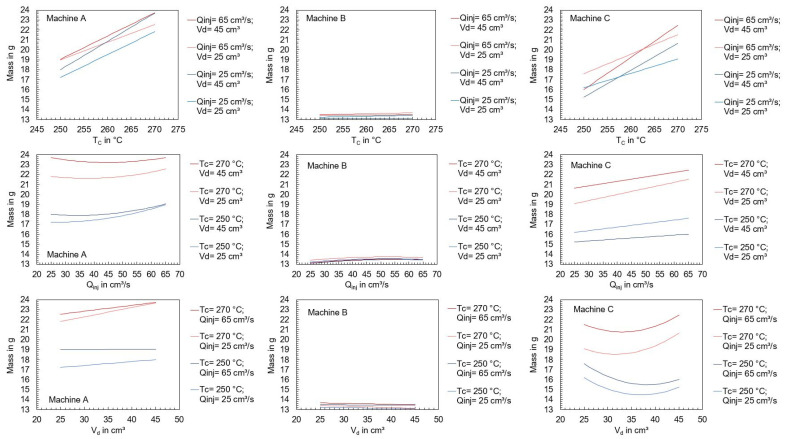
Effect of the machine setting parameters on the mean of the ejected polymer mass.

It is important to emphasize that the dosing and switchover volumes were set identically for each machine at the individual operating points. Theoretically, the masses ejected at the individual operating points should be the same, but this could not be observed. Figure 4 prominently illustrates significant differences in mass based on the operating point and the specific machine utilized. The results show that machines A and C show larger variations in ejected mass with changes in setting parameters than machine B. To visualize the individual effects of the setting parameters on the ejected polymer mass, regressions were carried out to create effect diagrams. The regression as well as the coefficient of determination are described in the section “Regression models” of Appendix A. The effect diagrams depending on the machine used are shown in Figure 5. Since the dosing and switchover volumes were varied by the same amount, only the dosing volume is used as representative of the screw stroke. The effect diagrams show that the mean ejected mass using machines A and C is strongly dependent on the set cylinder temperature, followed by the flow rate and the injection stroke. In comparison, changes in the operating point have hardly any effect on the ejected mass when machine B is used. This is surprising since machines A and B are hydraulic, and C is all-electric.

**Figure 6:**
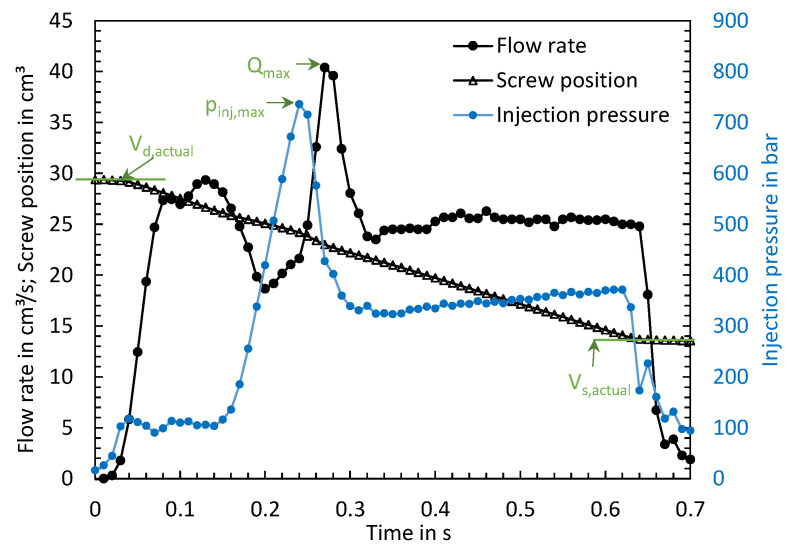
Exemplary representation of the time series of the flow rate, the screw position, and the injection pressure as well as characteristic values derived from it.

To be able to explain the reason for the strong variation in the ejected mass depending on the machine used, the time series of the process parameters were evaluated. For visualizing the multitude of time series and the influence of the machine-specific behavior on the time series of the process parameters, the max. flow rates, the max. injection pressures, and the actually achieved dosing as well as switchover volumes were calculated. Figure 6 visualizes the calculated characteristic values (green) using three exemplary time series of the flow rate, screw volume, and injection pressure.

**Figure 7:**
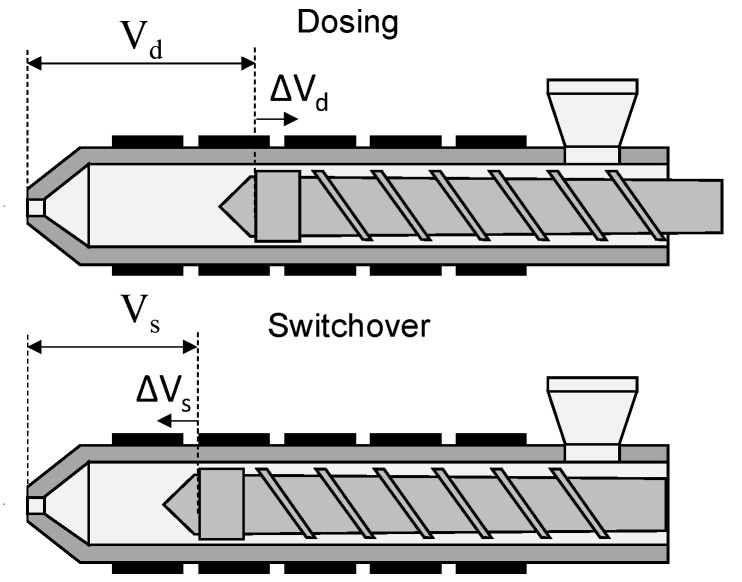
Plasticizing unit with screw position after dosing and at the switchover point.

To visualize the volumetric deviations, ΔV_d_ was calculated as the deviation of the screw position at the end of metering to the set dosing volume and ΔV_s_ as the deviation of the screw position at the end of injection to the set switchover volume. Figure 7 shows a schematic representation of the screw positions. Positive values of ΔV_d_ and ΔV_s_ lead to an increase in the injected volume.

The deviations of the volumes were calculated using the following formulas with V_i,target_ as the set volume and V_i,actual_ as the achieved volume. To determine the achieved volumes, the time series of the screw positions were evaluated (see Figure 6). V_d,actual_ was calculated using the maximum and V_s,actual_ using the minimum of the time series.

(1) $$\Delta V_{d}={\;V}_{d,\mathrm{target}}-{\;V}_{d,\mathrm{actual}}$$

(2) $$\Delta V_{s}=V_{s,\mathrm{actual}}-V_{s,\mathrm{target}}$$

(3) $$V_{\mathrm{inj}}=V_{d,\mathrm{actual}}-V_{s,\mathrm{actual}}$$

To convert the volumetric deviations into the deviation of the screw stroke Δs, the following formula was used.

(4) $$\Delta s=\frac{\Delta V}{4\pi \times {d_{\mathrm{screw}}}^{2}}$$

**Figure 8:**
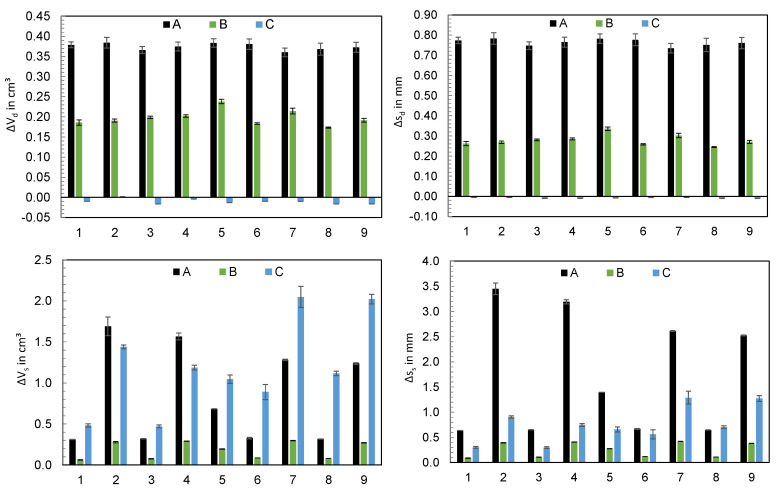
Deviations of the screw position from the set dosing volume (ΔV_d_) and switchover volume (ΔV_s_) as well as corresponding screw strokes s at the end of the injection phase w/o holding pressure (n = 6).

The computed values for the deviations of the dosing and switchover volumes as well as the screw strokes s calculated from these values are visualized in Figure 8. The values ΔV_d_ resp. Δs_d_ show that the hydraulic machines A and B meter more material than set, with machine A showing the highest deviations. In contrast, machine C meters almost exactly the set dosing volume. When considering ΔV_s_, machine B shows the greatest precision in approaching the switchover volume, with machines A and C exceeding the switchover point even more. Converted into screw stroke, machine A shows the strongest overrun in the majority of machine settings. 

**Figure 9:**
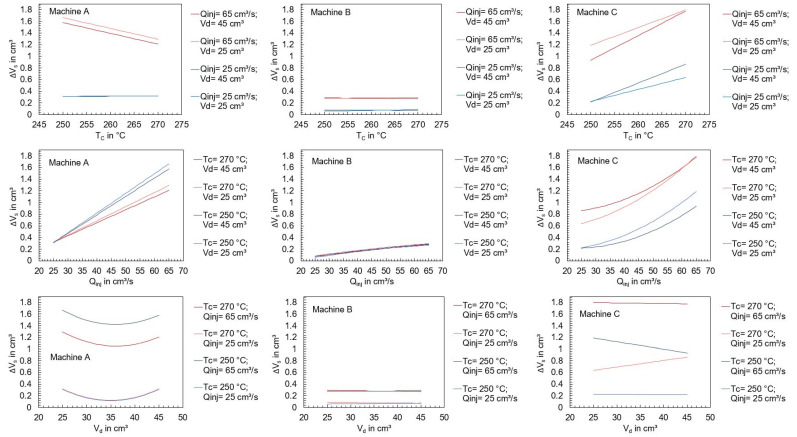
Effect of the machine setting parameters on the deviation of the screw position from the switchover volume.

A dependence of the operating point and the achieved dosing volume cannot be identified, which is not surprising since the selected operating points control the subsequent process phases, and the setting variables for parameterizing the metering phase were not varied. For this reason, the effects of the setting parameters are not evaluated further. In contrast, a significant influence of the setting parameters on the screw position during switchover is already evident. For the evaluation of these effects by using effect diagrams, regressions were carried out, which are described in the section “Regression models” of Appendix A. Figure 9 visualizes the mean of ΔV_s_ depending on the machine and the setting parameters. Thereby, a clear dependence on the respective machine setting becomes apparent: at high injection flow rates, the switchover point was overrun by the screw more than at low flow rates. This seems plausible because at a higher screw feed motion, the precision required to slow down the screw is lower. All other parameters of the experimental design show less influence on the overrun of the switchover volume, except for machine C, where increased cylinder temperatures led to a further overrun of the switchover point. Furthermore, it can be seen that in some cases, there are interactions between the setting parameters, which have an effect on the screw position at the end of the injection phase. For the interpretation of the results, the drive types are presented in more detail below.

**Figure 10:**
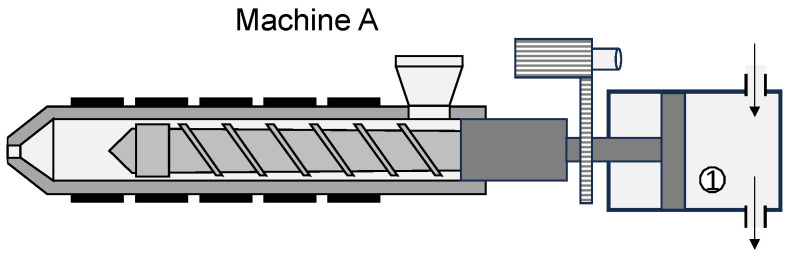
Draft of injection unit with the hydraulic system of machine A.

When considering the different machine designs, a variety of the effects observed can be attributed to the drive system and the respective control system. Machine A has an injection control system that pressurizes only the cylinder chamber that extends the hydraulic piston (number 1 shown in Figure 10).

When the switchover volume is reached, the hydraulic pressure is reduced and the valves (here: hydraulic 4-3 directional control valve) are switched off, which can result in a typical overrun of the switchover volume. The main drive, i.e., the flow and pressure control, is provided by a speed-controlled radial piston pump with analog control. On the one hand, the hydraulic pipes lead to a certain inertia, but on the other hand, the pump and the control loop also have a certain dynamic. Overshooting due to the I component of the PID-control is typical for this type of injection control.

**Figure 11:**
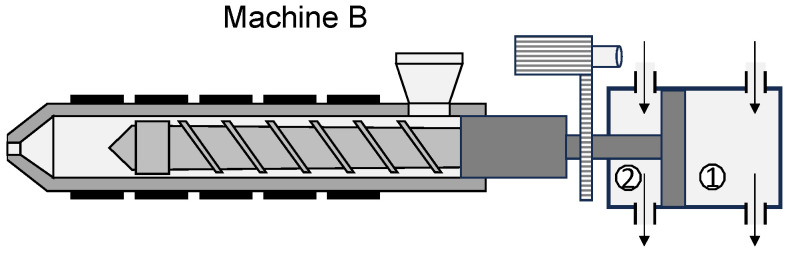
Draft of injection unit with the hydraulic system of machine B.

In contrast, machine B pressurizes both hydraulic chambers (number 1 and 2 shown in Figure 11) to adjust the position of the screw and is equipped with a position control system with a hydraulic accumulator.

A constant pump is responsible for charging the hydraulic accumulator. For this purpose, both cylinder chambers are actively controlled via servo-control valves. Since this control is faster than the control of machine A, the control parameters are adapted to the hydraulics accordingly. The position control results in a more precise control of the screw position.

Machine C differs significantly from machines A and B in terms of its type of drive. The position of the screw which is installed on machine C is controlled by an electric servo motor. Ring membrane sensors (eight strain gauge cells on each side of the cylinder) ensure the precise measurement of the position. It is typical with electric drives of this type that the machines can set the target injection flow rate very precisely. The braking of the screw, on the other hand, exhibits a certain amount of lag since the servo motor can only achieve a limited deceleration. 

After explaining the respective drive systems, the observed results can be explained as follows. The hydraulic machines meter more material, with machine A overrunning the switchover point the most due to its unidirectional hydraulic system. The servo motor of the electric machine meters the set dosing volume. The hydraulic injection molding machine with positioning control (machine B) enables the highest precision when approaching the switchover volume. This is due to its clamped system of hydraulics. The electric machine (machine C) shows an overrun of the switchover point due to the limited deceleration of the servo motor during braking. Here, both machines A and C show a clear dependence of the overrun characteristics on the flow rate and additionally for machine C on the cylinder temperatures. The screw position of machine B is not significantly influenced by the temperature or the screw stroke.

**Figure 12:**
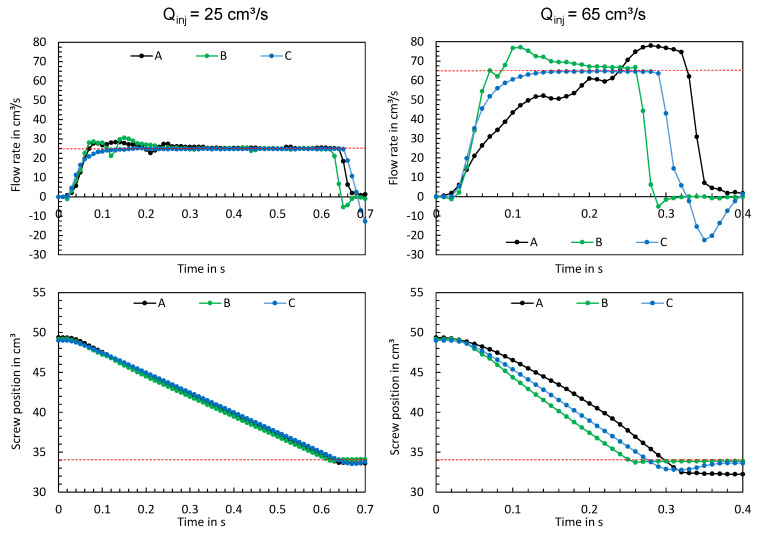
Measured flow rates and screw positions at test points 3 (**left**) and 4 (**right**).

In the following paragraphs, the characteristics of the time series and their significant differences will be examined in more detail. Figure 12 shows the time series measured when the machine was adjusted according to test points 3 and 4. The left side shows the time series at a flow rate of 25 cm^3^/s (test point 3), while the right side shows the time series at a flow rate of 65 cm^3^/s (test point 4). In the upper two diagrams, the red dashed lines represent the setting values of the flow rate and in the lower two diagrams the setting values of the switchover volume.

At low flow rates, all three machines reached the set flow rate, with machine C achieving a smooth asymptotic convergence to the set point. Machines A and B initially exceeded the set value and reached the value somewhat later after settling. In addition, the screw positions show that all three machines achieved an almost linear feed motion. At high injection flow rates, stronger differences between the time series become apparent. Machine C reaches the set injection flow rate asymptotically without exceeding it. In contrast, the hydraulic injection molding machines A and B show transient injection behavior, which can be seen from the fact that machine B exceeded the flow rate and machine A took a very long time to reach the set flow rate and then exceeded the set value. Likewise, the screw positions provide essential information on the feed movements of the screws. Machine B shows the fastest feed motion, with the switchover point being approached most precisely. Machine C, on the other hand, has a slower feed motion, but achieves the set flow rate more accurately. In addition, it can be seen from the time series of the screw position that machine C overruns the switchover point. Machine A shows the slowest feed motion, which remarkably exhibits a non-linear behavior. This is an indicator that machine A has a non-linear feed motion of the screw at high flow rate settings.

**Figure 13:**
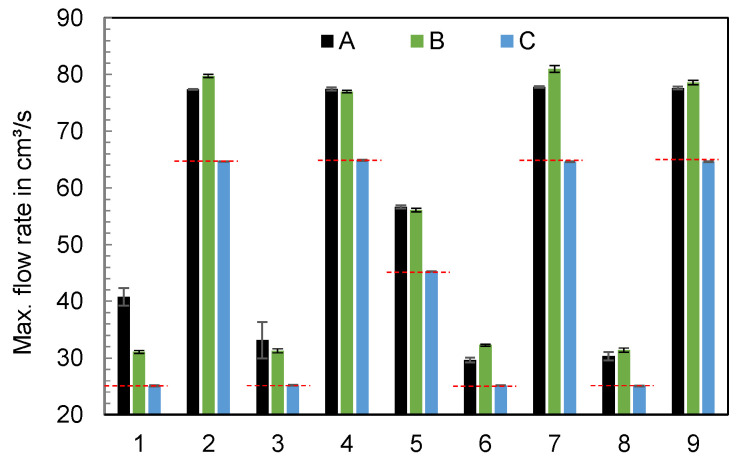
Maximum of the flow rates depending on the parameters of the experimental design and the machine (n = 6).

Figure 13 visualizes the maximum flow rate depending on the parameters of the experimental design. The set flow rates of the respective operating points are indicated by red dashed lines. The hydraulic machines A and B show a higher oscillation, especially at high flow rates. Furthermore, a dependence on the parameters of the experimental design can be seen for machines A and B, with machine C always reaching the set target value.

In summary, all three machines demonstrate different behavior during injection, which is influenced by the respective operating points.

**Figure 14:**
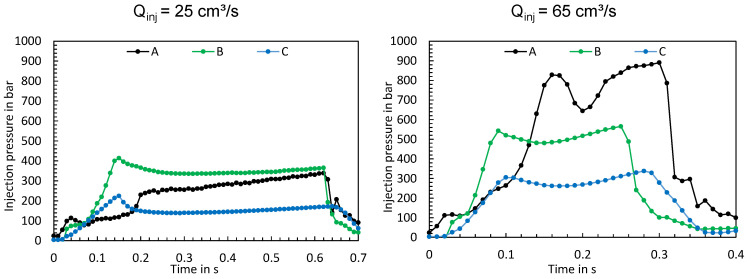
Measured injection pressures at test points 3 (**left**) and 4 (**right**).

In addition to the screw positions and flow rates, the resulting injection pressures were evaluated. The resulting injection pressures, two operating point examples of which are shown in Figure 14, show significant differences. It is apparent that the machine has a significant influence on the injection pressure profile depending on the respective operating point.

**Figure 15:**
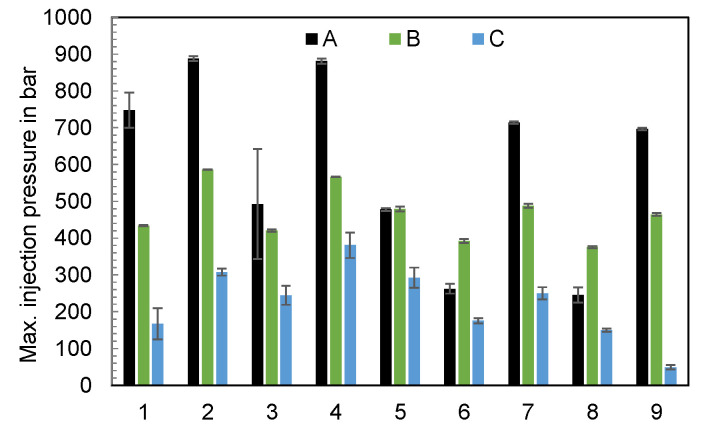
Maxima of the injection pressures depending on the parameters of the experimental design and the machine (n = 6).

The maxima of the injection pressure curves depending on the parameters of the experimental design, shown in Figure 15, demonstrate a clear dependence on the setting of the injection molding machine and also the machine type. The reasons why the injection pressures sometimes vary significantly (e.g., test points 1 and 3 when using machine A) may be due to material fluctuations or plugs.

**Figure 16:**
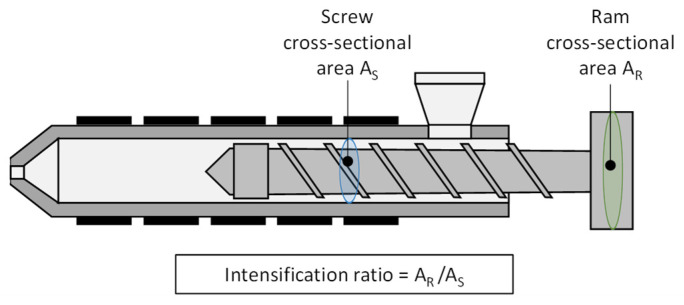
Representation of the screw and ram areas for the computation of the intensification ratio, in relation to [31].

One explanation for the difference in injection pressures is the intensification ratio (IR). The IR describes the pressure intensification between the hydraulic pressure applied and the specific pressure in the screw antechamber (Figure 16) [31]. Usually, the applied hydraulic pressure affects a larger cross-sectional area inside the piston than the specific pressure in the screw antechamber. As a result, a specific intensification ratio is obtained for each injection molding machine. 

Both hydraulic injection molding machines investigated in this work have an identical mounted injection unit. Consequently, the piston diameter for the hydraulics is also identical. Injection molding machine A has an IR of 15.4 with a screw diameter of 25 mm. Thus, a pressurization of, for example, one bar, results in a specific pressure of 15.4 bar in the screw antechamber. With a screw diameter of 30 mm, the injection molding machine B has an IR of 10.7. Consequently, a hydraulic pressure of one bar results in a specific pressure in the screw antechamber of 10.7 bar. For electrically driven injection molding machines, on the other hand, the pressure in the screw antechamber is identical to the pressure applied at the end of the screw. Accordingly, the IR ratio for electric injection molding machines is equal to one. Summing up, the IR factor is a relevant parameter that should be taken into account when creating a machine fingerprint.

### 3.3. Part C: Influence of the Material on the Machine-Specific Behavior of Two Different Hydraulic Injection Molding Machines

**Figure 17:**
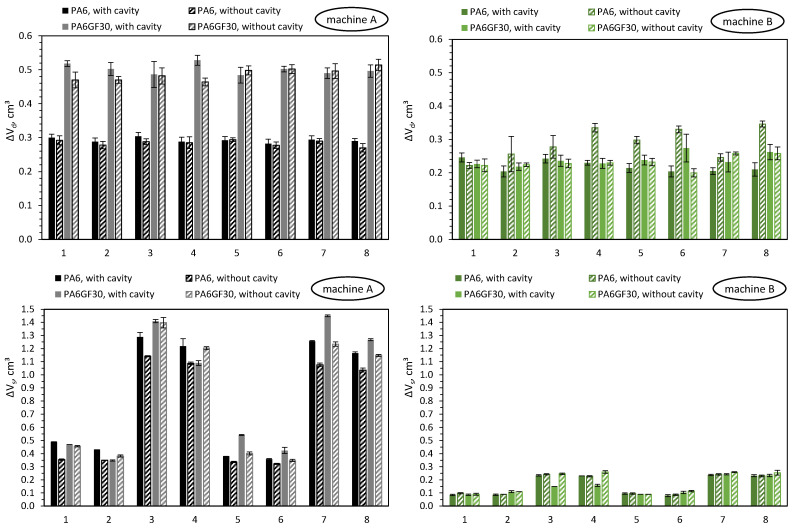
Deviations of the screw position from the set dosing volume (ΔV_d_) and switchover volume (ΔV_s_) when using unreinforced (PA) and glass-fiber-reinforced polyamide (PAGF30), when injecting into a cavity (with cavity) and into the atmosphere (without cavity).

In the following paragraphs, the focus will be exclusively on the machine-specific behavior of machines A and B when setting the dosing and switchover volumes while varying the polymer and the cavity. Figure 17 visualizes the measured deviations from the dosing volume ΔV_d_ and switchover volume ΔV_s_ when using unreinforced polyamide (PA) and reinforced polyamide with a glass-fiber content of 30% (PAGF30), when injected into a cavity (with cavity) and into the atmosphere (without cavity). 

**Figure 18:**
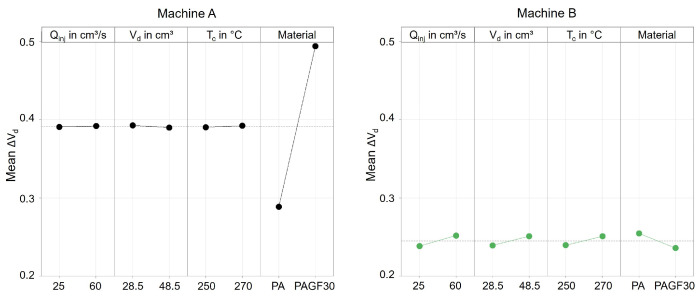
Effect of the machine setting parameters and the material on the mean deviation of the screw position from the dosing volume.

**Figure 19:**
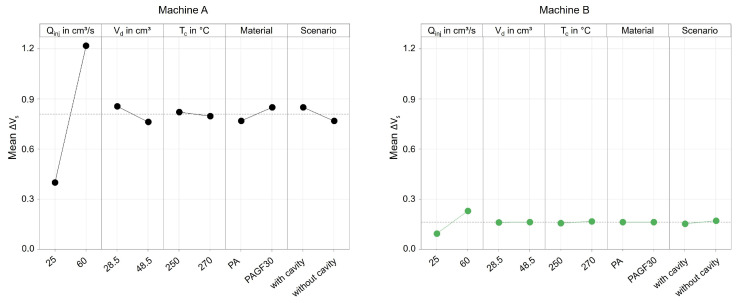
Effect of the machine setting parameters, the material, and the ejection into the atmosphere or cavity on the mean deviation of the screw position from the switchover volume.

Figure 17 already shows that there are significant differences between the machines when processing different materials. Due to the large number of the investigated influencing variables, the main effects are shown in Figure 18 and Figure 19. 

With machine A, the material has a significant influence on the precision for controlling the dosing volume. When processing PAGF30, the deviation ΔV_d_ was approximately 0.2 cm^3^ greater on average. When switching, no influence by the material can be seen, but instead there was a significant influence by the injection flow rate, which confirms the previously presented results. In comparison, machine B, in contrast to machine A, is not influenced at all by the setting parameters or the material. Merely an increase in the injection flow rate leads to a more extensive overrun of the switchover point. The results confirm that the position control of machine B results in a more precise control of the screw position, which has a much higher robustness against disturbance variables, i.e., even with changes in material properties or barrel temperatures.

The main finding from this is that material changes with respect to various melt volume rates (see Table 7) have different effects on the machine-specific behavior of the machine when controlling the screw positions. Despite the same size of their plasticizing units, hydraulic machines can exhibit very different behavior depending on the machine components and their kind of control. Machine B provides more reproducible results, which is due to the clamped hydraulic system and presumably the control strategy for regulating the screw positions. In contrast, machine A shows significant changes in the amount of material metered when the material changes. This inaccuracy of the screw during backward movement during metering is due to the de-pressurization of just a single hydraulic chamber. The results also illustrate that the presented material-dependent behavior of the machines should be investigated and taken into account when developing a machine fingerprint of the machine.

## 4. Conclusions

Within the scope of these studies, the behavior of two hydraulic IMMs of different sizes and one electric IMM were compared. In retrospect to the research questions formulated at the beginning, the following results can be summarized:

- During the initial machine start-up phase, distinct variations in machine behavior were observed. These variations were assessed by analyzing the ejected polymer mass from shot to shot. The primary insight gained from this investigation is that the machine has a significant influence on the start-up behavior and the duration until a reproducible process characterized by mass stability is achieved. Sampling at the beginning of an experiment can thus be superposed by machine-specific thermal transient processes.
- Variations in the operating points of the machines revealed substantial disparities in the process outcomes, specifically in terms of ejected polymer mass. It has been found that individual changes in the operating point settings have different effects depending on the machine used.
- To elucidate the underlying causes of the substantial disparities in the process outcomes, an evaluation of the measured time series flow rate, injection pressure, and screw position was conducted. By using the time series, the transient behavior of the injection flow rate, the switchover point overrun, and the linearity of the screw’s feed motion was assessed. It was shown that significant differences in the measured time series occur, which can be attributed to the machine, the components of the respective machine, and also the machine setting parameters. Accordingly, the time series of the process parameters offer detailed information for comparing the performance of different machines.
- In order to assess the impact of material characteristics on machine behavior, unreinforced as well as glass fiber-reinforced polyamide was processed on two different hydraulic injection molding machines. The time series of the process parameters have shown that, in addition to the operating point, the material properties also have an effect on the precision of the machine. The machine’s ability to compensate material fluctuations depends on the machine itself. The material-specific influence on the precision of the machine is especially of particular interest in the processing of recycled material, as variations in the material characteristics have different effects on the process. In this context, knowledge about the robustness of the machines to material variations can be very helpful in the selection of the MMMC. For this purpose, the measured time series can be utilized for evaluating the robustness of the machine.
- The above-described occurrences can be responsible for discrepancies between CFD simulation results, which do not account for the machine’s dynamic behavior, and experimental data. This is a crucial consideration, particularly when using simulation data as a substitute for experimental results.

The results highlight the considerable impact that the machine can exert on the process and its outcomes. Especially when comparing simulations and experiments, this can lead to significant divergences. Looking ahead to further research, the development of a machine fingerprint is recommended. The machine fingerprint can contribute to the transfer of knowledge from simulation to experiment, to the evaluation of the similarity of different MMMCs, and to the transfer of data from one machine to another. This machine fingerprint should encompass the following scenarios:

- The thermal start-up behavior of the machines should be characterized in more detail. Here, monitoring of the hydraulic oil temperature, temperature sensors installed in the mold, or measuring sensors in the screw antechamber could potentially be used to identify relevant factors with an impact on the start-up dynamics.
- The high-resolution time series of the process parameters provide a lot of information about the behavior of the machine. The fingerprint should include characteristics describing the machine-dependent dynamics of the process parameters. Characteristic values of the machine control (e.g., of the PID controller) could be used to obtain information on the transient behavior of the injection flow rate or characteristics describing the dynamics of the hydraulics and its components as well as the overrun characteristics of the servo motor. In addition, the IR is an important parameter which provides information about the relationship between hydraulic pressure and pressure in the screw antechamber, and should be taken into account.
- To incorporate the material-specific characteristics of the machines into the fingerprint, reference tests could be established, for example. Using an inline viscosity nozzle (as presented in [32]), the correlations between material properties and process behavior could be examined for generating specific coefficients.

These investigations show the need to analyze the machine-specific behavior in order to describe and understand the differences between the processes when using various MMMCs. It is recommended to analyze a variety of machines using higher complexity experimental designs in further studies to be able to derive universal models. 

## Figures and Tables

**Table 1:** Series of experiments.

Part	IMM	Materials	Mold	Machine Setup	Focus
A	A, B, and C	Unfilled polyamide (PA)	Closed	Constant operating point	Influence of the machine on the process result during start-up
B	A, B, and C	Unfilled polyamide (PA)	Closed	Variation in nine operating points	Influence of the machine at varying operating points
C	A and B	Unfilled (PA) and glass fiber-reinforced polyamide (PAGF30)	Closed and open (injection into atmosphere)	Variation in eight operating points	Influence of the material and mold

**Table 2:** Injection molding machine data.

	A	B	C
Clamping force, kN	500	1100	1500
Max. injection flow rate, cm^3^/s	66	136	174
Screw diameter d_screw_, mm	25	30	45
Nozzle diameter, mm	4	3	3
Max. injection pressure, bar	2500	2000	2470
Calculated stroke volume, cm^3^	59	85	318
Effective screw length (length/diameter), -	24	20	22
Drive, -	HM		EM

**Table 3:** Operating point during start-up, Part A.

Designation	Q_inj_, cm^3^/s	V_d_, cm^3^	V_s_, cm^3^	T_c_, °C
0	45	35	24	260

**Table 4:** Experimental design, Part B.

Designation	Q_inj_, cm^3^/s	V_d_, cm^3^	V_s_, cm^3^	T_c_, °C
1	25	25	14	250
2	65	25	14	250
3	25	45	34	250
4	65	45	34	250
5	45	35	24	260
6	25	25	14	270
7	65	25	14	270
8	25	45	34	270
9	65	45	34	270

**Table 5:** Experimental design, Part C.

Designation	Q_inj_, cm^3^/s	V_d_, cm^3^	V_s_, cm^3^	T_c_, °C
1	25	30.5	12.5	250
2	25	50.5	32.5	250
3	60	30.5	12.5	250
4	60	50.5	32.5	250
5	25	30.5	12.5	270
6	25	50.5	32.5	270
7	60	30.5	12.5	270
8	60	50.5	32.5	270

**Table 6:** Constant injection molding machine parameters.

Parameter	Parts A and B	Part C
Screw peripheral speed, m/min	18	15
Back pressure, bar	60	75
Decompression volume, cm^3^	4	2
Mold temperature, °C	75	60
Cooling time (with cavity), s	15	30

**Table 7:** Description of the material used.

	PA	PAGF30
Trade name	Ultramid B3S	Ultramid B3EG6
Glass fiber ratio, %	0	30
Melt volume rate, cm^3^/10 min	160	35
Density, g/cm^3^	1.13	1.36
Tensile modulus, MPa	3500	9500
Breaking elongation, %	4	3.5

## Data Availability

Data are contained within the article.

## Appendix A

**Table A1:** Settings of the cylinder zone temperatures depending on the machine used.

Machine	Zone 1 (T_c_)	Zone 2	Zone 3	Zone 4	Zone 5	Zone 6	Zone 7	Zone 8
A	250 °C	245 °C	240 °C	235 °C	230 °C			
B	250 °C	245 °C	240 °C	235 °C	230 °C			
C	250 °C	245 °C	245 °C	240 °C	240 °C	235 °C	235 °C	230 °C

### Regression Models

Table A3 lists the regression equations for calculating the mass depending on the variables shown in Table A2. The quality of the regression equation was described by the coefficient of determination R^2^ [33]. The regressions were used to visualize the effect diagrams shown in Figure 5.

**Table A2:** Variables to describe the mass.

Variable	Parameter
X1	Q_inj_ in cm^3^/s
X2	V_d_ in cm^3^
X3	T_c_ in °C

**Table A3:** Regression to describe the mass depending on the variables.

Machine	Regression	R^2^
A	mass in g = −31.57 + 0.2815 X1 − 0.622 X2 + 0.1927 X3 + 0.001228 X1^2^ − 0.000881 X1 × X2 − 0.001265 X1 × X3 + 0.002732 X2 × X3	98.43%
B	mass in g = 8.283 + 0.04272 X1 + 0.0727 X2 + 0.01741 X3 − 0.000436 X1^2^ + 0.000120 X1 × X2 − 0.000334 X2 × X3	94.65%
C	mass in g = 40.41 − 0.2668 X1 − 2.422 X2 − 0.0445 X3 + 0.01180 X2^2^ − 0.000786 X1 × X2 + 0.001287 X1 × X3 + 0.006272 X2 × X3	98.16%

In addition, Table A4 lists the regression equations for describing ΔV_s_, which represents the deviation of the screw position from the switchover volume. The regressions were used to visualize the effect diagrams shown in Figure 9.

**Table A4:** Regression to describe ΔV_s_ depending on the variables.

Machine	Regression	R^2^
A	ΔV_s_ in cm^3^ = −1.401 + 0.15407 X1 − 0.1357 X2 + 0.01212 X3 + 0.001974 X2^2^ − 0.000103 X1 × X2 − 0.000471 X1 × X3	99.38%
B	ΔV_s_ in cm^3^ = −1.1013 + 0.01362 X1 + 0.01990 X2 + 0.003719 X3 − 0.000039 X1^2^ − 0.000013 X1 × X2 − 0.000017 X1 × X3 − 0.000075 X2 × X3	99.80%
C	ΔV_s_ in cm^3^ = 0.39 − 0.0654 X1 − 0.1387 X2 − 0.00014 X3 + 0.000402 X1^2^ − 0.000308 X1 × X2 + 0.000244 X1 × X3 + 0.000584 X2 × X3	98.84%
